# Circulating immune index predicting the prognosis of patients with hepatocellular carcinoma treated with lenvatinib and immunotherapy

**DOI:** 10.3389/fonc.2023.1109742

**Published:** 2023-02-23

**Authors:** De-Zhen Guo, Shi-Yu Zhang, San-Yuan Dong, Jia-Yan Yan, Yu-Peng Wang, Ya Cao, Sheng-Xiang Rao, Jia Fan, Xin-Rong Yang, Ao Huang, Jian Zhou

**Affiliations:** ^1^ Department of Liver Surgery and Transplantation, Liver Cancer Institute, Zhongshan Hospital, Fudan University, Key Laboratory of Carcinogenesis and Cancer Invasion (Fudan University), Ministry of Education, Shanghai, China; ^2^ Shanghai Key Laboratory of Organ Transplantation, Zhongshan Hospital, Fudan University, Shanghai, China; ^3^ Department of Radiology, Zhongshan Hospital, Fudan University, Shanghai Institute of Medical Imaging, Shanghai, China; ^4^ Cancer Research Institute, Central South University, Key Laboratory of Carcinogenesis and Cancer Invasion, Ministry of Education, Changsha, China; ^5^ Institute of Biomedical Sciences, Fudan University, Shanghai, China; ^6^ State Key Laboratory of Genetic Engineering, Fudan University, Shanghai, China

**Keywords:** hepatocellular carcinoma, prognostic models, immunotherapy, lenvatinib, circulating immune index

## Abstract

**Background:**

Immune checkpoint inhibitor (ICI)-based combination therapy has opened a new avenue for the treatment of multiple malignancies including hepatocellular carcinoma (HCC). However, considering the unsatisfactory efficacy, biomarkers are urgently needed to identify the patients most likely to benefit from ICI-based combination therapy.

**Methods:**

A total of 194 patients undergoing ICI-based combination therapy for unresectable HCC were retrospectively enrolled and divided into a training cohort (n = 129) and a validation cohort (n = 65) randomly. A novel circulating immune index (CII) defined as the ratio of white blood cell count (×10^9^/L) to lymphocyte proportion (%) was constructed and its prognostic value was determined and validated.

**Results:**

Patients with CII ≤ 43.1 reported prolonged overall survival (OS) compared to those with CII > 43.1 (median OS: 24.7 vs 15.1 months; 6-, 12-, 18-month OS: 94.2%, 76.7%, 66.1% vs 86.4%, 68.2%, 22.8%, *P* = 0.019), and CII was identified as an independent prognostic factor for OS (hazard ratio, 2.24; 95% confidence interval, 1.17-4.31; *P* = 0.015). These results were subsequently verified in the validation cohort. Additionally, patients with low CII levels had improved best radiological tumor response (complete response, partial response, stable disease, progressive disease: 3%, 36%, 50%, 11% vs 0%, 27%, 46%, 27%; *P* = 0.037) and disease control rate (89% vs 73%; *P* = 0.031) in the pooled cohort and better pathologic response (pathologic complete response, major pathologic response, partial pathologic response, no pathologic response: 20%, 44%, 28%, 8% vs 0%, 0%, 40%, 60%; *P* = 0.005) in the neoadjuvant cohort. Detection of lymphocyte subsets revealed that an elevated proportion of CD4+ T cells was related to better OS, while the proportion of CD8+ T cells was not.

**Conclusions:**

We constructed a novel circulating immune biomarker that was capable of predicting OS and therapeutic efficacy for HCC patients undergoing ICI and lenvatinib combination therapy.

## 1 Introduction

Hepatocellular carcinoma (HCC) is the sixth most frequently diagnosed malignancy and the third leading cause of cancer-related death globally (1). Despite improvements in screening programs and diagnostic tools, patients were frequently diagnosed as advanced tumor stage and not eligible for curative treatments such as surgical resection, liver transplantation, or ablation, leading to a poor prognosis (2, 3).

Immune checkpoint inhibitor (ICI), predominantly represented by monoclonal antibodies to programmed death 1 (PD-1) or programmed death ligand 1 (PD-L1), has opened a new avenue for cancer therapy and has been approved as a treatment for multiple solid tumor types including advanced HCC (4–8). However, the objective response rates of ICI monotherapy only range from 14% to 20% in HCC, whereas all the treated confront the risk of several immune-related side effects that sometimes are life-threatening (2, 9). Recently, ICI-based combination therapy such as ICI plus angiogenesis inhibitors or tyrosine kinase inhibitors (TKI) becomes a promising regimen and achieves great efficacy in various malignancies, including HCC (10–12). However, the LEAP-002 trial which applied ICI and lenvatinib failed to reach the primary endpoint, indicating that not all patients could benefit from it. Therefore, biomarkers capable of predicting the prognosis and efficacy of ICI-based combination therapy before treatment initiation are urgently needed.

It is well established that the tumor immune microenvironment affected the efficacy of immunotherapy (13, 14). Considering the tumor samples from the biopsy are hardly available from advanced HCC patients, it is more feasible to screen biomarkers from peripheral blood. Recently, emerging evidence has shown that circulating immune status might partially reflect the tumor immune microenvironment and serve as a potential prognostic biomarker of immunotherapy in various tumors (15–17). In HCC, traditional tumor marker alpha-fetoprotein (AFP) or well-established predicting models including neutrophil to lymphocyte ratio (NLR) and platelet to lymphocyte ratio (PLR), as well as newly constructed scores such as C-reactive protein and AFP in Immunotherapy (CRAFITY) have been reported to be correlated with prognosis of patients treated with immunotherapy (18–20). However, the association between circulating immune status and efficacy of ICI and lenvatinib combination therapy in HCC was not clear.

Here, we constructed a novel circulating immune index (CII) based on the immune components in peripheral blood and validated its prognostic value for predicting efficacy and outcomes in patients with unresectable HCC treated with ICI and lenvatinib combination therapy. Meanwhile, the prognostic values of different subtypes of immune cells in peripheral blood were also explored.

## 2 Materials and methods

### 2.1 Patient inclusion

Patients with HCC who underwent combination therapy of ICI (nivolumab, camrelizumab, sintilimab, pembrolizumab, toripalimab, atezolizumab, or tislelizumab) and lenvatinib for unresectable HCC at Zhongshan Hospital between February 2018 and December 2020 were retrospectively enrolled as the combination therapy cohort. Meanwhile, a cohort of patients with HCC receiving neoadjuvant combination therapy and subsequent surgical resection at the same institute from February 2019 to September 2021 was recruited as the neoadjuvant cohort. The inclusion criteria for patients were listed as follows: (1) histopathological or radiological diagnosis of HCC; (2) without a history of other malignancies; (3) availability of complete clinicopathological features and follow-up data; (4) at least one radiological evaluation after the initiation of treatment. Patients without measurable intrahepatic foci were excluded from the analysis.

Clinicopathological information, radiological data, and laboratory parameters at baseline (within 30 days before the start of therapy) were collected. The tumor stage was classified under the Barcelona Clinic Liver Cancer (BCLC) staging system (21). Liver function was evaluated based on the Child-Pugh score (22). Approval for this study was granted by the Ethics Committee of Zhongshan Hospital (No. B2020-401) and informed consent was obtained from each patient included in the analysis.

### 2.2 Assessment of efficacy

Each patient was followed up every 2 to 4 weeks after the initiation of treatment until loss to follow-up or death. Laboratory tests including routine blood, serum AFP, and liver function were conducted at each follow-up visit. Dynamic contrast-enhanced abdominal magnetic resonance imaging (MRI) or computed tomography (CT) scans were performed about every 3 months or when intrahepatic tumor progression was suspected. And extrahepatic metastases were assessed using chest CT, isotope bone scan, positron emission tomography-computed tomography (PET-CT), and other radiological examinations. The best radiological tumor response was evaluated as per the Response Evaluation Criteria in Solid Tumors version 1.1 (RECIST1.1). The treatment response was evaluated independently by experienced radiologists. The primary endpoint was overall survival (OS), which was defined as the interval from the initiation of treatment to death from any cause. The secondary endpoint was the disease control rate (DCR), defined as the proportion of complete response (CR), partial response (PR), and stable disease (SD). The time of last follow-up was October 1st, 2022.

### 2.3 Circulating immune index

The CII was defined as the ratio of white blood cell (WBC) count (×10^9^/L) to lymphocyte proportion (%). The optimal threshold of CII was calculated using the X-tile software (Version 3.6.1, Yale University, New Haven, CT). Ultimately, 43.1 was determined as the optimal cutoff value of CII in the training cohort and was used to stratify patients in subsequent analyses.

### 2.4 Assessment of histopathologic response to combination therapy

For the patients in the neoadjuvant cohort, tissue specimens obtained from surgical resection were handled and sampled according to the guidelines for pathological diagnosis of HCC (23). Hematoxylin and eosin (H&E)-stained sections from these specimens were histologically assessed *via* experienced pathological experts, based on the immune-related pathologic response criteria (irPRC) (24). Pathologic complete response (pCR) was defined as the absence of any viable tumor in the tumor bed area. Patients with residual viable tumor (RVT) were stratified into three groups: major pathologic response (MPR, RVT% ≤ 10%), partial pathologic response (pPR, 10% < RVT% < 90%), and no pathologic response (pNR, RVT% ≥ 90%) (25). Neoadjuvant combination therapy failure was defined as reaching pPR or pNR.

### 2.5 Statistical analysis

Statistical analyses were conducted using R software (Version 4.1.1, R Foundation for Statistical Computing, Vienna, Austria) and IBM SPSS Statistics (Version 26.0, IBM Corp, Armonk, NY). Continuous variables were presented as mean (standard deviation) or median (interquartile range). Differences between the two groups were analyzed by T-test or Mann-Whitney U test as appropriate. For discrete variables, proportions were calculated, and the Chi-square test or Fisher’s exact test was applied for intergroup comparisons. Survival analysis was conducted by the Kaplan-Meier method, and the differences between the two groups were compared using the log-rank test. The Cox regression model was applied to perform univariate and multivariate analyses. The hazard ratio (HR) and 95% confidence interval (CI) were calculated. Variables with a *P* value inferior to 0.1 in univariate analysis were included for multivariate analysis. All the statistical tests were two-tailed, and a *P* value < 0.05 was considered statistically significant.

## 3 Results

### 3.1 Patient characteristics

**Figure 1:**
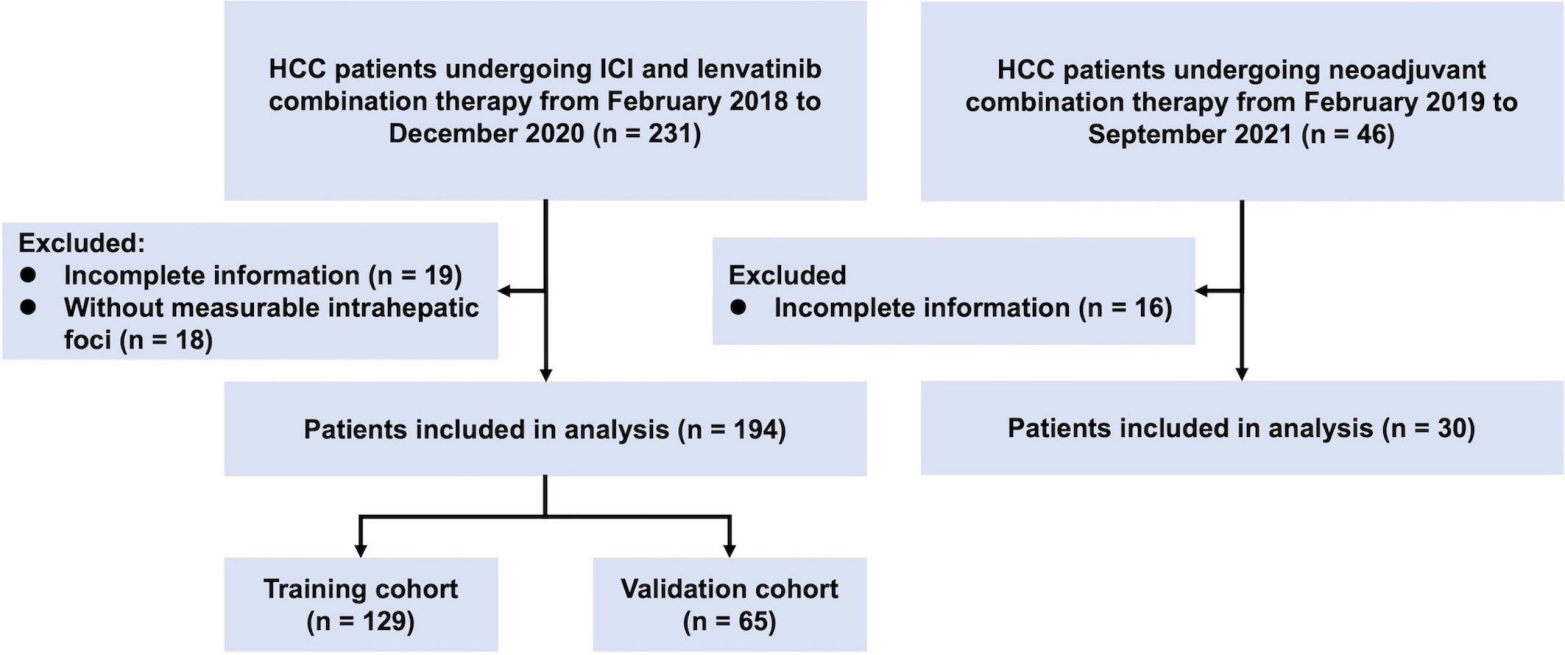
Flowchart of the recruitment pathway for patients. HCC, hepatocellular carcinoma; ICI, immune checkpoint inhibitor.

A total of 194 patients with unresectable HCC undergoing ICI and lenvatinib combination therapy were retrospectively enrolled in the study (**Figure 1**). All patients were divided into the training cohort (n = 129) and the validation cohort (n = 65) randomly. There was no significant difference in baseline characteristics between the training cohort and the validation cohort (**Table 1**). The majority of patients were hepatitis B surface antigen (HBsAg) positive (86%), BCLC C stage (84%), and Child-Pugh grade A (91%). 125 (64%) patients harbored macrovascular invasion and 71 (37%) had extrahepatic metastases. The median duration of follow-up was 14.2 (1.1-38.1) months. 72 (37%) patients died at the end of the follow-up.

**Table 1:** Clinicopathological characteristics in the training cohort and validation cohort.

Variables	Levels	Training cohort	Validation cohort	*P* value
Case		129	65	
Age, years	≤50	45 (35%)	27 (42%)	0.365
	>50	84 (65%)	38 (58%)	
Gender	female	15 (12%)	4 (6%)	0.226
	male	114 (88%)	61 (94%)	
HBsAg	negative	21 (16%)	7 (11%)	0.303
	positive	108 (84%)	58 (89%)	
Child-Pugh stage	A	116 (90%)	61 (94%)	0.362
	B	13 (10%)	4 (6%)	
BCLC stage	B	23 (18%)	8 (12%)	0.322
	C	106 (82%)	57 (88%)	
Lines of combination therapy	first	117 (91%)	58 (89%)	0.746
	later	12 (9%)	7 (11%)	
Tumor number	solitary	43 (33%)	27 (42%)	0.261
	multiple	86 (67%)	38 (58%)	
Tumor size, cm	≤5	42 (33%)	16 (25%)	0.254
	>5	87 (67%)	49 (75%)	
Macrovascular invasion	no	47 (36%)	22 (34%)	0.722
	yes	82 (64%)	43 (66%)	
Extrahepatic metastases	no	82 (64%)	41 (63%)	0.947
	yes	47 (36%)	24 (37%)	
AFP, ng/mL	≤400	65 (50%)	33 (51%)	0.960
	>400	64 (50%)	32 (49%)	
Best radiological response	CR	2 (2%)	3 (5%)	0.589
	PR	48 (37%)	19 (29%)	
	SD	62 (48%)	33 (51%)	
	PD	17 (13%)	10 (15%)	

HBsAg, hepatitis B surface antigen; BCLC, Barcelona Clinic Liver Cancer; AFP, alpha-fetoprotein; CR, complete response; PR, partial response; SD, stable disease; PD, progressive disease.

### 3.2 High CII associates with poor OS in the training cohort

**Figure 2:**
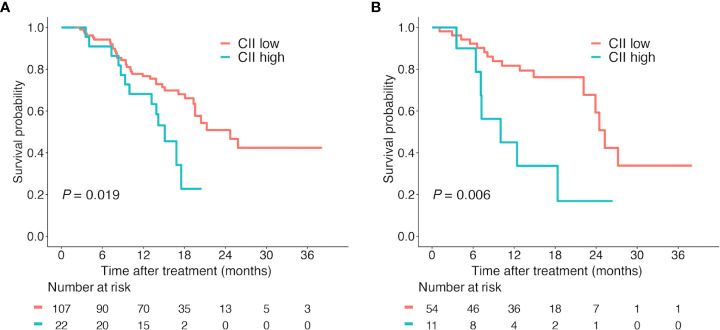
Kaplan-Meier survival curves according to CII. Overall survival according to CII (cutoff value = 43.1) in the training cohort **(A)** and validation cohort **(B)**. Abbreviations: CII, circulating immune index.

Patients in the training cohort were separated into the CII low group (n = 107) and CII high group (n = 22) according to the cutoff value of 43.1. The Kaplan-Meier survival analysis was utilized to evaluate the prognostic value of CII. CII low group showed prolonged OS compared to CII high group (median OS: 24.7 vs 15.1 months; *P* = 0.019; **Figure 2A**). The 6-, 12-, and 18-month cumulative survival rates were 94.2%, 76.7%, and 66.1% for patients with low CII respectively, compared with 86.4%, 68.2%, and 22.8% for patients with high CII.

Univariate Cox regression analysis revealed that the presence of extrahepatic metastases and high CII levels were associated with poor OS (**Table 2**). Upon the following multivariate analysis, extrahepatic metastases (HR, 1.95; 95%CI, 1.11-3.42; *P* = 0.021; **Table 2**) and CII (HR, 2.24; 95%CI, 1.17-4.31; *P* = 0.015; **Table 2**) were identified as independent prognostic factors for OS in HCC patients receiving ICI and lenvatinib combination therapy.

**Table 2:** Univariate and multivariate analyses for overall survival in the training cohort and validation cohort.

Variables	Training cohort				Validation cohort			
	Univariate analysis		Multivariate analysis		Univariate analysis		Multivariate analysis	
	HR (95%CI)	*P* value	HR (95%CI)	*P* value	HR (95%CI)	*P* value	HR (95%CI)	*P* value
Age, years (≤50: >50)	0.72 (0.40-1.29)	0.271			1.83 (0.75-4.48)	0.184		
Gender (female: male)	0.87 (0.39-1.93)	0.727			1.13 (0.15-8.49)	0.906		
HBsAg (negative: positive)	1.45 (0.68-3.11)	0.337			1.57 (0.44-5.63)	0.489		
Child-Pugh stage (A: B)	1.87 (0.66-5.28)	0.238			1.96 (0.45-8.65)	0.372		
Lines of combination therapy (first: later)	2.03 (0.91-4.54)	0.084	2.19 (0.98-4.93)	0.058	3.22 (1.16-8.91)	0.024	2.68 (0.92-7.80)	0.070
Tumor number (solitary: multiple)	1.50 (0.81-2.77)	0.197			0.87 (0.38-1.98)	0.744		
Tumor size, cm (≤5: >5)	1.25 (0.69-2.26)	0.458			1.84 (0.67-5.02)	0.233		
Macrovascular invasion (no: yes)	1.29 (0.72-2.32)	0.387			0.66 (0.29-1.51)	0.321		
Extrahepatic metastases (no: yes)	1.88 (1.07-3.30)	0.028	1.95 (1.11-3.42)	0.021	2.38 (1.04-5.46)	0.040	2.09 (0.88-4.95)	0.094
AFP, ng/mL (≤400: >400)	1.36 (0.77-2.40)	0.284			0.72 (0.31-1.66)	0.441		
CII (≤43.1: >43.1)	2.14 (1.12-4.11)	0.022	2.24 (1.17-4.31)	0.015	3.27 (1.33-8.07)	0.010	3.27 (1.33-8.07)	0.010
NLR (≤2.7: >2.7)	1.39 (0.79-2.46)	0.257			1.09 (0.48-2.48)	0.839		
PLR (≤ 33.1: >133.1)	1.00 (0.56-1.79)	0.998			0.88 (0.37-2.07)	0.761		
SII (≤330: >330)	1.46 (0.81-2.63)	0.212			1.08 (0.46-2.57)	0.855		

HR, hazard ratio; CI, confidence interval; HBsAg, hepatitis B surface antigen; AFP, alpha-fetoprotein; CII, circulating immune index; NLR, neutrophil to lymphocyte ratio; PLR, platelet to lymphocyte ratio; SII, systemic immune-inflammation index.

### 3.3 Verification of the prognostic value of CII in the validation cohort

In the validation cohort, 65 patients were grouped in the same way (CII low group, n = 54; CII high group, n = 11). Consistent with the results from the training cohort, survival analysis indicated that low CII levels were significantly correlated with better OS (median OS: 25.3 vs 10.0 months; *P* = 0.007; **Figure 2B**). The cumulative survival rates of the CII low group were markedly superior to the CII high group at 6, 12, and 18 months (92.3%, 81.7%, 76.2% vs 78.8%, 33.8%, 16.9%). Furthermore, univariate and multivariate analyses were performed to validate the independent prognostic role of CII (HR, 3.27; 95%CI, 1.33-8.07; *P* = 0.010; **Table 2**) for OS in patients with HCC.

### 3.4 The prognostic significance of CII in different subgroups

**Figure 3:**
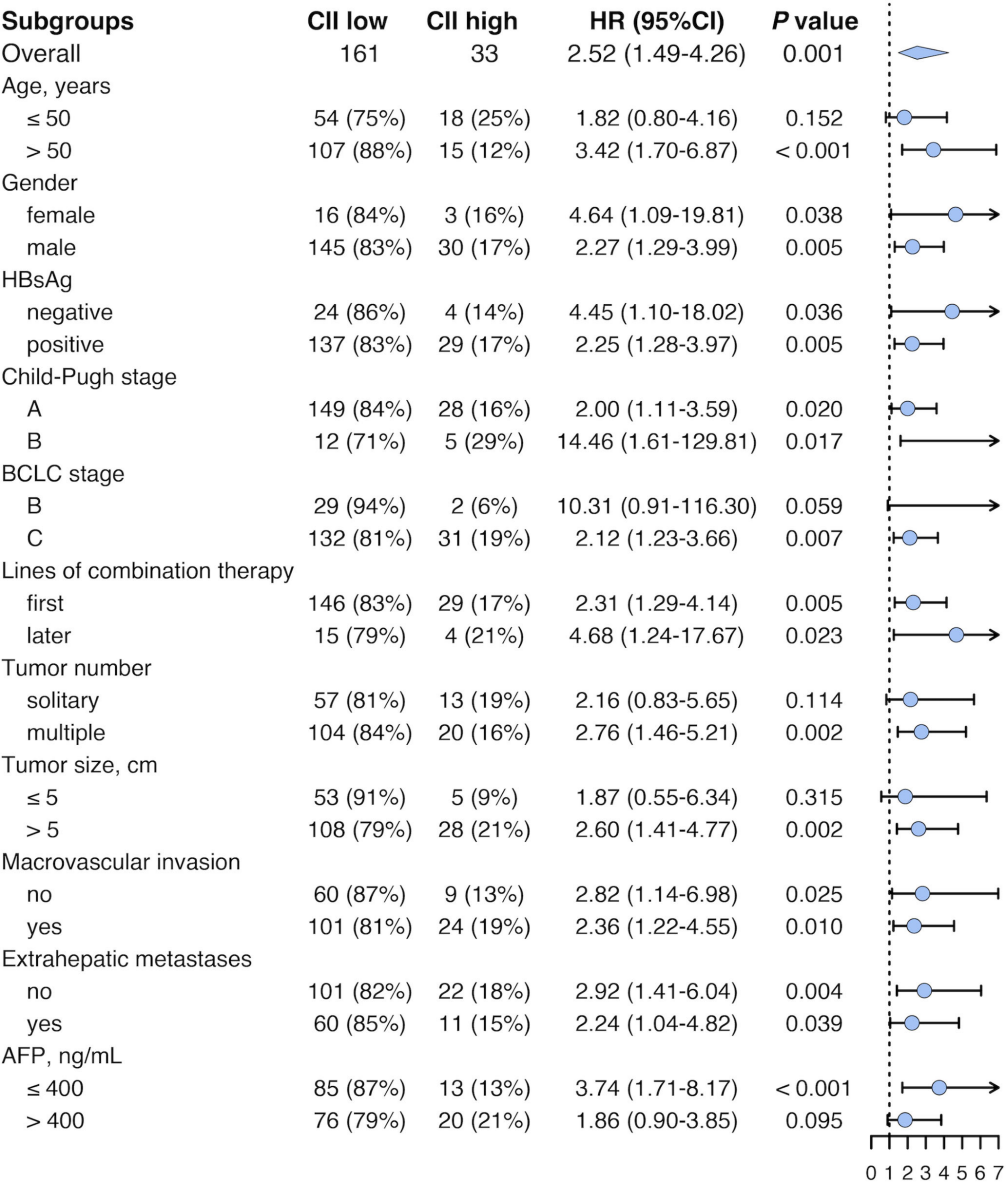
Forest plot of HR for survival in different subgroups in the pooled cohort. HR, hazard ratio; CII, circulating immune index; CI, confidence interval; HBsAg, hepatitis B surface antigen; BCLC, Barcelona Clinic Liver Cancer; AFP, alpha-fetoprotein.

In the pooled cohort, subgroup analyses were performed to explore the applicability of our CII in patients with diverse characteristics (**Figure 3**). Median OS was 24.7 (95% CI 21.3-28.1) months for CII low group (n = 161) and 14.2 (95% CI 11.0-17.3) months for CII high group (n = 33) (*P* = 0.001). In patients who underwent combination therapy as first-line treatment, median OS was 25.3 (95% CI 21.1-29.4) months for CII low group (n = 146), and 15.1 (95% CI 11.6-18.6) months for CII high group (n = 29) (*P* = 0.005). Similarly, in patients who underwent combination therapy as later-line treatment, median OS was 17.1 (95% CI 2.4-31.9) months for CII low group (n = 15), and 4.1 (95% CI 0.6-7.6) months for CII high group (n = 4) (*P* = 0.023). Comparable results were observed in other subgroups including female and male, HBsAg negative and positive, Child-Pugh stage A and B, first and later lines of combination therapy, absence and presence of macrovascular invasion, absence and presence of extrahepatic metastases, age > 50 years, BCLC stage C, multiple tumors, tumor size > 5 cm, and AFP ≤ 400 ng/mL.

### 3.5 CII predicts the best radiological response in the pooled cohort

To investigate the predictive value of CII for efficacy, we compared the best radiological tumor response of the two groups in the pooled cohort. The result demonstrated that high CII levels were concerned with unsatisfactory radiological response to ICI and lenvatinib combination therapy. In CII low group, the patients assessed as CR, PR, SD, and progressive disease (PD) were 5 (3%), 58 (36%), 80 (50%), and 18 (11%), respectively. As a comparison, 0 (0%), 9 (27%), 15 (46%), and 9 (27%) patients had CR, PR, SD, and PD in CII high group, respectively (*P* = 0.037; **Table 3**). The DCR was significantly higher in CII low group (89%) than that in CII high group (73%) (*P* = 0.031; **Table 3**).

**Table 3:** Efficacy according to CII in the pooled combination therapy cohort and neoadjuvant cohort.

Pooled combination cohort	CII low, n=161	CII high, n=33	*P* value
Best radiological response			0.037
CR	5 (3%)	0 (0%)	
PR	58 (36%)	9 (27%)	
SD	80 (50%)	15 (46%)	
PD	18 (11%)	9 (27%)	
Disease control			0.031
Yes (CR/PR/SD)	143 (89%)	24 (73%)	
No (PD)	18 (11%)	9 (27%)	
Neoadjuvant cohort	CII low, n=25	CII high, n=5	*P* value
Pathologic response			0.005
pCR	5 (20%)	0 (0%)	
MPR	11 (44%)	0 (0%)	
pPR	7 (28%)	2 (40%)	
pNR	2 (8%)	3 (60%)	
Neoadjuvant treatment failure			0.014
Yes (pPR/pNR)	9 (36%)	5 (100%)	
No (pCR/MPR)	16 (64%)	0 (0%)	

CII, circulating immune index; CR, complete response; PR, partial response; SD, stable disease; PD, progressive disease; pCR, pathologic complete response; MPR, major pathologic response; pPR, partial pathologic response; pNR, no pathologic response.

### 3.6 CII predicts pathologic response in the neoadjuvant cohort

To explore the predictive value of CII pathologically, a total of 30 HCC patients who received neoadjuvant ICI and lenvatinib combination therapy were included and their resection specimens were histologically assessed. Baseline characteristics were displayed in **Supplementary Table 1**. In CII low group, 5 (20%), 11 (44%), 7 (28%), and 2 (8%) patients had pCR, MPR, pPR, and pNR, respectively, while 2 (40%) and 3 (60%) patients had pPR and pNR in CII high group (*P* = 0.005; **Table 3**). And the patients assessed as neoadjuvant treatment failure were 9 (36%) vs 5 (100%) for CII low group vs CII high group (*P* = 0.014; **Table 3**).

### 3.7 Association of circulating lymphocyte subsets with the efficacy of ICI and lenvatinib combination therapy

To further investigate the circulating lymphocyte subsets associated with the efficacy of ICI and lenvatinib combination therapy, we performed flow cytometry in 77 patients in the pooled cohort. Among all the detected lymphocyte subsets, significant differences between the CII groups were observed in the proportion of CD4+ T cells. Patients with low CII levels showed a higher proportion of CD4+ T cells than patients with high CII levels (*P* = 0.044, **Table 4**).

**Table 4:** Lymphocyte subsets according to CII in the pooled cohort.

Variables	CII low, n=65	CII high, n=12	*P* value
Proportion of CD4+ T cells, %	40.0 (35.7-45.8)	34.1 (31.1-40.3)	0.044
Proportion of CD8+ T cells, %	24.5 (20.4-29.9)	29.4 (23.8-31.8)	0.209
Proportion of CD3+ T cells, %	70.6 (64.0-75.8)	67.4 (62.5-77.2)	0.569
Proportion of B cells, %	12.1 (8.3-15.9)	10.4 (8.4-12.2)	0.347
Proportion of NK cells, %	15.4 (10.3-22.1)	20.1 (12.2-24.4)	0.350

CII, circulating immune index.

**Figure 4:**
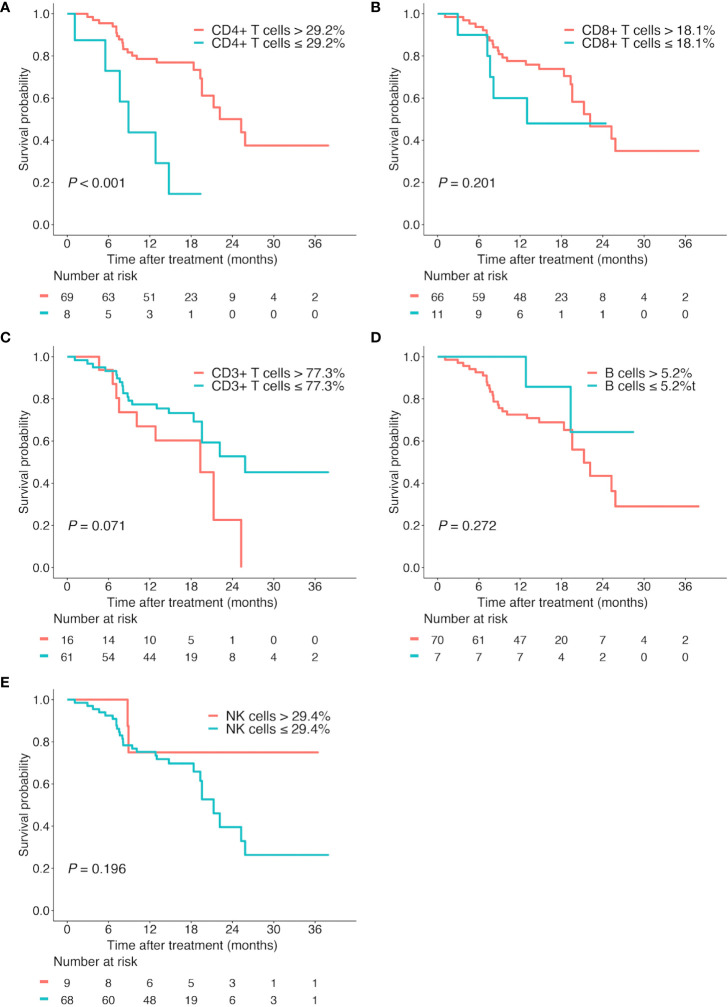
Kaplan-Meier survival curves according to different lymphocyte subsets. Overall survival according to the proportion of CD4+ T cells **(A)**, CD8+ T cells **(B)**, CD3+ T cells **(C)**, B cells **(D)**, and NK cells **(E)**.

Then, we performed the Kaplan-Meier analyses for these lymphocyte subsets based on their optimal cutoff thresholds. The high proportion of CD4+ T cells (CD4+ T cells > 29.2%) was found to prolong OS (median OS, 25.3 vs 8.9 months; *P* < 0.001; **Figure 4A**), while the proportion of CD8+ T cells, CD3+ T cells, B cells, and NK cells showed no significant association with OS (all *P* > 0.05, **Figures 4B–E**).

## 4 Discussion

Increasing studies have revealed the prognostic significance of circulating immune biomarkers in patients with various types of cancer receiving immunotherapy. However, the lack of such biomarkers in HCC makes it difficult to distinguish patients suitable for ICI-based immunotherapy. In the current study, a simple biomarker CII was developed based on the easily accessible WBC count and lymphocyte proportion. It was identified as an independent prognostic factor for OS in patients with HCC undergoing ICI and lenvatinib combination therapy. Patients with CII ≤ 43.1 reported prolonged OS compared to those with CII > 43.1. These results were subsequently verified in a validation cohort. Concerning the efficacy prediction, patients with low CII levels had improved radiological tumor response and DCR in the pooled cohort. The predictive value for treatment efficacy was further validated in a neoadjuvant cohort by assessing the pathologic response of resection specimens.

There were some prognostic models for predicting the survival of patients who received systemic treatment for advanced HCC, including sorafenib and immunotherapy. The PROSASH and PROSASH-II model, which consisted of serum albumin, bilirubin, AFP, macrovascular invasion, extrahepatic spread, and largest tumor size, could predict the survival of patients with HCC treated with sorafenib (26, 27). The CRAFITY score, which was based on the baseline level of AFP and C-reactive protein, could stratify HCC patients undergoing immunotherapy into three groups with significantly different treatment responses and OS (20). Another study investigated the prognostic significance of the systemic inflammatory response in HCC patients receiving ICI, finding that high levels of NLR and PLR were associated with shorter OS and progression-free survival (PFS) (28). However, all these studies were designed for TKI or ICI monotherapy and their efficiency in TKI and ICI combination therapy needs further investigation. Few studies investigated the clinical significance of circulating immune status for efficacy and prognosis of immunotherapy. In the present study, we found that the combination of WBC count and lymphocyte proportion was associated with outcomes after ICI and lenvatinib combination therapy for unresectable HCC and could serve as a biomarker to identify patients with potential survival benefits. For the first time, we found the association between CII and the efficacy of ICI-based combination therapy in HCC, indicating that the circulating immune status could reflect the immune microenvironment in tumors and predict the efficacy of ICI-based combination therapy.

The lymphocyte is one of the most commonly applied hematological indices to evaluate the systemic immune status. A variety of biomarkers based on the lymphocyte were initially proved to be involved with the efficacy and prognosis of ICI-based immunotherapy in various tumors, especially in lung cancer (15, 16, 28–30). For example, Lung Immune Prognostic Index, the combination of derived neutrophil to lymphocyte ratio and lactate dehydrogenase, was proved to be correlated with unfavorable outcomes for immunotherapy, but not for chemotherapy (29). Besides, low absolute lymphocyte count was found to be associated with shorter OS and PFS in patients with non-small cell lung cancer treated with nivolumab (9). In HCC, although lymphocyte is a predominant component of prognostic models predicting outcomes after radical resection or TACE, such as NLR, PLR, and systemic immune-inflammation index (SII) (31, 32), its prognostic value in immunotherapy, especially in ICI and lenvatinib combination therapy was not clear. In our study, the prognostic value of circulating lymphocytes was expanded into ICI-based combination therapy in unresectable HCC, which makes it possible to dynamically monitor the efficacy of combination therapy through circulating immune status in the future.

Although ICI facilitates anti-tumor immune responses by activating various immune cells of both the innate and adaptive arms, lymphocytes are the linchpins of ICI-based immunotherapy (33). Given that ICI-induced lymphocyte subset changes can be reflected in peripheral blood, flow cytometry which depicts the pre-treatment circulating immune status of patients may help to further explain the prognostic value of CII. CD8+ T cells have been classically viewed as the predominant effector responsible for anti-tumor immune responses due to its direct tumor-killing feature, while CD4+ T cells function as auxiliary roles by promoting the priming and differentiation of naive CD8+ T cells (33, 34). Emerging studies indicate that heightened levels of CD4+ T cells and CD8+ T cells in the peripheral blood before or after immunotherapy are connected with improved clinical outcomes (33, 35). However, we found that it was CD4+ T cells instead of CD8+ T cells that correlated with outcomes after ICI and lenvatinib combination therapy. The high proportion of CD4+ T cells before treatment predicted higher response and longer OS for patients. To our knowledge, it was the first time that the prognostic value of circulating T cells, specifically CD4+ T cells had been revealed in ICI-based combination therapy in unresectable HCC, which might help clinical decision-making. And increasing the proportion of CD4+ T cells in peripheral blood may be a potential way to improve the efficacy of combination therapy.

The tumor immune microenvironment serves a pivotal role in tumor metastasis, relapse, and treatment resistance (36). In HCC, the immune microenvironment composed of various immune and stromal cells is characterized by strong immunosuppressive (37). Liver-resident macrophages, M2-type tumor-associated macrophages, regulatory T cells, and myeloid-derived suppressor cells are the predominant immunosuppressive cells that contributed to the immune escape of HCC through the expression of immunosuppressive factors, especially checkpoint molecules (38, 39). Thus, immunotherapy represented by ICI has captured increasing attention in HCC. However, due to the high heterogeneity of HCC, ICI monotherapy did not show improvement in OS compared with sorafenib, along with limited objective response rates (2, 40). To overcome the resistance of immunotherapy, the combination of ICI and angiogenesis inhibitors or TKI was proposed and its efficacy was evaluated in patients with unresectable HCC. Regrettably, ICI plus lenvatinib failed to improve OS and PFS compared with lenvatinib monotherapy in the LEAP-002 trial, which is the standard first-line treatment for unresectable HCC. Therefore, except for the accurate identification of candidate patients who may benefit from current treatment regimens, efforts should be made to reveal the heterogeneity of HCC and develop novel therapeutic agents and combination strategies.

There were some limitations of the current study. The most prominent is the retrospective design since this is subject to unintentional biases. Moreover, the sample size was relatively small due to the short period of combination therapy for HCC. Thus, a prospective study with a large sample size was needed to validate the prognostic model. Additionally, all patients involved in our study were from China and the most common etiology was hepatitis B virus. The efficiency of the model in other ethnicities and etiologies needs further validation. Besides, the components of CII are both easily accessible clinical hematological indices. However, against the backdrop of the rapid development of precision medicine and the high heterogeneity of HCC, more individualized approaches for prognosis stratification based on genomic information need to be explored.

## 5 Conclusion

In conclusion, our study identified the ratio of WBC count to lymphocyte proportion as a novel circulating immune biomarker that was capable of predicting OS and therapeutic efficacy for HCC patients undergoing ICI and lenvatinib combination therapy. Application of CII may help to distinguish patients expected to benefit from ICI and lenvatinib combination therapy and guide treatment decisions.

## Data availability statement

The raw data supporting the conclusions of this article will be made available by the authors, without undue reservation.

## Ethics statement

The studies involving human participants were reviewed and approved by the Ethics Committee of Zhongshan Hospital. The patients/participants provided their written informed consent to participate in this study. 

## Author contributions

JZ, AH, X-RY, and D-ZG conceptualized this study and wrote the manuscript. S-YZ and S-YD conducted data analysis. J-YY and Y-PW collected the clinical information of patients. YC, S-XR, and JF interpreted the data. All authors edited the manuscript. All authors contributed to the article and approved the submitted version.

## Glossary

**Table:**

HCC	hepatocellular carcinoma
ICI	immune checkpoint inhibitor
PD-1	programmed death 1
PD-L1	programmed death ligand 1
TKI	tyrosine kinase inhibitor
CII	circulating immune index
BCLC	Barcelona Clinic Liver Cancer
AFP	alpha-fetoprotein
MRI	magnetic resonance imaging
CT	computed tomography
PET-CT	positron emission tomography-computed tomography
RECIST1.1	Response Evaluation Criteria in Solid Tumors version 1.1
OS	overall survival
DCR	disease control rate
CR	complete response
PR	partial response
SD	stable disease
WBC	white blood cell
H&E	hematoxylin and eosin
irPRC	immune-related pathologic response criteria
pCR	pathologic complete response
RVT	residual viable tumor
MPR	major pathologic response
pPR	partial pathologic response
pNR	no pathologic response
HR	hazard ratio
CI	confidence interval
HBsAg	hepatitis B surface antigen
PD	progressive disease
NLR	neutrophil to lymphocyte ratio
PLR	platelet to lymphocyte ratio
PFS	progression-free survival
SII	systemic immune-inflammation index
