# Supplementary material for: Circulating immune index predicting the prognosis of patients with hepatocellular carcinoma treated with lenvatinib and immunotherapy
Source: Front Oncol. 2023 Feb 23;13:1109742. doi: 10.3389/fonc.2023.1109742 (PMC9997675; doi:10.3389/fonc.2023.1109742)
Supplement: Supplementary file 1 [file Table_1.docx]

Supplementary Material

**Supplementary Table 1. Clinicopathological characteristics in the neoadjuvant cohort.**

| **Variables** | **Levels** | **Neoadjuvant cohort** |
| --- | --- | --- |
| Case |  | 30 |
| Age, years | ≤50 | 7 (23%) |
|  | >50 | 23 (77%) |
| Gender | female | 4 (13%) |
|  | male | 26 (87%) |
| HBsAg | negative | 6 (20%) |
|  | positive | 24 (80%) |
| Child-Pugh stage | A | 28 (93%) |
|  | B | 2 (7%) |
| BCLC stage | A | 6 (20%) |
|  | B | 4 (13%) |
|  | C | 20 (67%) |
| Tumor number | solitary | 17 (57%) |
|  | multiple | 13 (43%) |
| Tumor size, cm | ≤5 | 6 (20%) |
|  | >5 | 24 (80%) |
| Macrovascular invasion | no | 10 (33%) |
|  | yes | 20 (67%) |
| AFP, ng/mL | ≤400 | 15 (50%) |
|  | >400 | 15 (50%) |

Abbreviations: HBsAg, hepatitis B surface antigen; BCLC, Barcelona Clinic Liver Cancer; AFP, alpha-fetoprotein.
